# Phosphate supplementation for hypophosphatemia during continuous renal replacement therapy in adults

**DOI:** 10.1080/0886022X.2018.1561374

**Published:** 2019-03-26

**Authors:** Young-Hye Song, Eun-Hye Seo, Yang-Sook Yoo, Young-Il Jo

**Affiliations:** a Dialysis Center, Konkuk University Medical Center, Seoul, Korea;; b Department of Cellular and Molecular Medicine, Konkuk University School of Medicine, Seoul, Korea;; c College of Nursing, The Catholic University of Korea, Seoul, Korea;; d Division of Nephrology, Konkuk University School of Medicine, Seoul, Korea

**Keywords:** Hypophosphatemia, phosphate, continuous renal replacement therapy, supplementation, hyperphosphatemia

## Abstract

**Background:** Hypophosphatemia is common during continuous renal replacement therapy (CRRT) in critically ill patients and can cause generalized muscle weakness, prolonged respiratory failure, and myocardial dysfunction. This study aimed to investigate the efficacy and safety of adding phosphate to the dialysate and replacement solutions to treat hypophosphatemia occurring in intensive CRRT in critically ill patients.

**Methods:** We retrospectively analyzed 73 patients treated with intensive CRRT (effluent flow ≥35 ml/kg/hr) in the intensive care unit. The control group (group 1, *n* = 22) received no phosphate supplementation. The treatment groups received dialysate and replacement solution phosphate supplementation at 2.0 mmol/L (group 2, *n* = 26) or 3.0 mmol/L (group 3, *n* = 25).

**Results:** The CRRT-induced hypophosphatemia incidence was 59.0%. Correction of hypophosphatemia with phosphate supplementation changed the mean serum phosphorus levels to 1.24 ± 0.37 and 1.44 ± 0.31 mmol/L in groups 2 and 3, respectively (*p* = .02). The time required for correction was 1.65 ± 0.80 and 1.39 ± 1.43 days for groups 2 and 3, respectively and was significantly longer in group 2 (*p* = .02). After supplementation, hypophosphatemia, and hyperphosphatemia both occurred in 7% of group 2. Group 3 developed no hypophosphatemia, but 20% developed hyperphosphatemia. The serum phosphate levels in hyperphosphatemia cases returned to normal within 2.0 days (group 2) and 1.0 day (group 3) after stopping phosphate supplementation.

**Conclusion:** Phosphate supplementation effectively corrected CRRT-induced hypophosphatemia in critically ill patients with an acute kidney injury. The use of 2 mmol/L phosphate is appropriate in patients with CRRT-induced hypophosphatemia, but a different concentration could be required to prevent hypophosphatemia at the start of CRRT.

## Introduction

In the intensive care unit (ICU), continuous renal replacement therapy (CRRT) plays a crucial role in treating acute kidney injury (AKI) accompanying severe electrolyte imbalance or acid-base disorder. However, complications may occur during CRRT, including hypophosphatemia, various types of electrolyte imbalances, acid-base imbalances, hypotension, infection, bleeding, and hypothermia [1,2]. The incidence of hypophosphatemia during CRRT is wide-ranging across studies, from 27 to 78% [3–6]. Hypophosphatemia is relatively tolerable in chronically ill patients but can cause respiratory muscle failure and prolonged respiratory failure in critically ill patients, if severe [7]. Moreover, hypophosphatemia is reported to be an independent predictor of mortality in some patient groups such as patients with sepsis [8]. Therefore, the prevention and treatment of hypophosphatemia in CRRT patients is highly important. Much research has been conducted on the prevention of hypophosphatemia and adding phosphate to dialysate and replacement solution has been found to be relatively effective [9–11]. However, even if commercialized phosphate-containing dialysis solutions (e.g., Phoxillium^®^) are used, CRRT-induced hypophosphatemia cannot be completely prevented [12,13].

The likelihood of hypophosphatemia occurrence is greater in intensive CRRT, as the CRRT duration is longer and the CRRT dose is higher [14–16]. According to two high-quality multi-center randomized controlled trials (RCTs) conducted to compare intensive CRRT (prescribed dose over 35 mL/kg/hr) and less intensive CRRT (conventional dose), an increase of CRRT dose to 35–40 mL/kg/hr induced a higher frequency of hypophosphatemia, but did not increase survival [17,18]. Based on such study results, recent clinical practice guideline (CPG) has recommended an adequate CRRT delivered a dose of 20–25 mL/kg/hr [19]. However, in some patients like postsurgical AKI patients, intensive CRRT may reduce the risk of mortality [20]. A higher dose CRRT may also help some patients in a hypercatabolic state [21]. For instance, severe metabolic acidosis patients (pH <7.1) may temporarily require an effluent flow rate >40 mL/kg/hr for effective correction of acidosis. However, it is not known how best to correct hypophosphatemia occurring in such intensive CRRT.

The purpose of the present study was to evaluate the efficacy and safety of phosphate supplementation used to treat hypophosphatemia occurring during intensive CRRT in critically ill patients.

## Materials and method

We conducted a retrospective analysis of 73 AKI patients who underwent CRRT at the Konkuk University Medical Center ICU between 2009 and 2013. Only patients over 18 years of age were included. The exclusion criteria were as follows: a prescribed dose of CRRT <35 mL/kg/hr, a duration of CRRT <72 h, the presence of hypophosphatemia before CRRT, the presence of end-stage renal disease (ESRD) requiring dialysis before the ICU stay, and a treatment history of intravenous supplementation of phosphate during the ICU stay. The patients were divided into three groups based on phosphate supplementation, comprising the control group (group 1) and two treatment groups (group 2 and 3). The control group (*n* = 22) included patients with no phosphate supplementation. Treatment groups included patients in whom 2.0 mmol/L phosphate was supplemented to dialysate and replacement solution (group 2, *n* = 26), and patients in whom 3.0 mmol/L phosphate was supplemented (group 3, *n* = 25). All clinical and laboratory data were collected from the electrical medical record (EMR) database of the research hospital.

Hypophosphatemia treatment protocols changed during the course of the study. During the first year of the study period, phosphate supplementation was provided via intravenous route for severe hypophosphatemia only. Mild and moderate hypophosphatemia were closely observed without providing phosphate supplementation (Period 1). From the second year on, if patients presented with mild hypophosphatemia, phosphate was added to dialysate and replacement solutions (Period 2). For phosphate supplementation, Phosten^®^ (JW Pharmaceutical, Seoul, Korea) was used, a monobasic potassium phosphate product containing 619.09 mg (20 mmol) of phosphate and 780 mg (20 mmol) of potassium (K^+^) per 20 mL vial. The phosphorus level of the dialysate and replacement solution was 2 mmol/L or 3 mmol/L. If the serum phosphorus level measured in the morning was 1.78 mmol/L or higher, phosphate supplementation was not provided. Hypophosphatemia was defined as mild (<0.81 mmol/L, <2.50 mg/dL), moderate (<0.61 mmol/L, <1.90 mg/dL), or severe (<0.32 mmol/L, <1.00 mg/dL), and hyperphosphatemia was defined as serum phosphorus levels >1.78 mmol/L.

CRRT was administered using PRISMA CRRT equipment (Gambro, Lundia AB, Lund, Sweden) and ST 100 filter (Gambro, 1.0 m^2^, AN69 membrane). Hemosol B0 solution (Gambro) was used as a dialysate and replacement fluid solution (Supplementary Table 1). The prescribed effluent flows ranged from 30–35 mL/kg/hr based on the patients’ weight before the onset of AKI. In cases of hypercatabolic state and severe hyperkalemia or metabolic acidosis, the prescribed dose was 35 mL/kg/hr. The blood flow rate was maintained at 150 mL/min or higher. Heparin or Nafamostat mesilate was used as an anticoagulant depending on the prescription from the physician. In all patients, serum analytes (P, Ca, Na, K, albumin, lactate, etc.) were regularly measured during CRRT. Serum levels of P, Ca, and K were checked daily at 5 AM and before CRRT. Additional blood testing was performed, if necessary. The biochemistry tests were performed by the Diagnostic Testing Department of Konkuk University Medical Center, using TBA 200FR-neo (TOSHIBA, Otawara-shi, Japan) and reagents manufactured by SEKISU (Tokyo, Japan). The reference ranges used for serum P, Ca, and K levels were 0.81–1.78, 2.0–2.7, and 3.5–5.5 mmol/L, respectively.

The study was approved by the Human Research Ethics Committee at Konkuk University Medical Center (No. KUH1010350) and registered online with the Clinical Research Information Service (CRIS) (N. KCT0001341).

### Variables

The endpoint was the incidence of hypophosphatemia or hyperphosphatemia following phosphate supplementation. Additionally, the incidence of CRRT-induced hypophosphatemia, the time for hypophosphatemia to occur during CRRT, serum phosphorus level after it returned to normal following phosphate supplementation, and CRRT-induced changes in serum K and Ca were examined.

### Statistical analysis

Data are reported as mean ± SD. Statistical analysis was performed using IBM SPSS version 22 (IBM Inc., Armonk, NY). Comparison of categorical variables was performed by chi-square test or Fisher’s exact test. Continuous variables were analyzed by Student *t*-test or Mann–Whitney *U*-test. The means of the three groups were compared using analysis of variance (ANOVA). All analyses were 2-tailed. *p* values <.05 were considered statistically significant.

## Results

A total of 73 patients with AKI requiring CRRT were included in the final analysis. The baseline demographics and characteristics of the patients are summarized in Table 1. The mean age for patients of the study cohort was 65.05 ± 12.22 years, and 40 patients (54.8%) were male. The effluent flow prescribed was 35 mL/kg/hr. The total CRRT running time for all patients was 12877.5 h and the downtime period was 3303.0 h, accounting for 25.6% of the total treatment period. The patients were divided into 3 groups for analysis. Group 1 (control group, *n* = 22) included patients with no phosphate supplementation during Period 1. Group 2 (treatment group with 2.0 mmol/L phosphate, *n* = 26) included patients in whom 2.0 mmol/L phosphate was supplemented to dialysate and replacement solution during Period 2, and group 3 (treatment group with 3.0 mmol/L phosphate, *n* = 25) included patients in whom 3.0 mmol/L phosphate was supplemented during Period 2. The patients were divided into three groups for analysis. Group 1 (control group, *n* = 22) included patients with no phosphate supplementation during Period 1. Group 2 (treatment group with 2.0 mmol/L phosphate, *n* = 26) included patients in whom 2.0 mmol/L phosphate was supplemented to dialysate and replacement solution during Period 2, and group 3 (treatment group with 3.0 mmol/L phosphate, *n* = 25) included patients in whom 3.0 mmol/L phosphate was supplemented during Period 2.

**Table 1.** Clinical characteristics of the patients at the time of initiation of continuous renal replacement therapy (CRRT).

	Group 1 (*n* = 22)	Group 2 (*n* = 26)	Group 3 (*n* = 25)	*X*^2^*/F*	*p*
	*N* (%) / mean ± standard deviation				
Sex					
Male	13 (59.1)	11 (42.3)	16 (64.0)	0.91	.89
Female	9 (40.9)	15 (57.7)	9 (36.0)		
Age (years)	65.85 ± 11.20	66.65 ± 12.34	62.65 ± 13.12	0.48	.63
Number of patients on ventilators	16 (72.7)	12 (46.1)	16 (64.0)	2.46	.12
ICU duration (days)	31.55 ± 20.41	45.65 ± 50.23	33.45 ± 22.65	1.30	.29
APACHE II score	21.83 ± 4.49	24.80 ± 6.53	27.71 ± 6.55	1.59	.24
Duration of CRRT (days)	6.12 ± 3.15	7.73 ± 3.46	6.76 ± 3.24	1.45	.26
Down time of CRRT (hrs)	43.32 ± 54.20	46.5 ± 55.25	45.76 ± 55.57	0.02	.97
Risk for refeeding syndrome	5 (22.7)	3 (11.5)	4 (16.0)	0.53	.59
Severe sepsis	9 (40.9)	15 (57.6)	11 (44.0)	0.02	.97
Underlying disease					
Cardiovascular disease	6 (27.3)	9 (34.6)	2 (8.0)	0.65	.88
Renal disease	5 (22.7)	5 (19.2)	9 (36.0)		
Liver disease	2 (9.1)	2 (7.7)	2 (8.0)		
Gastrointestinal disease	1 (4.5)	0 (0.0)	2 (8.0)		
Others	8 (33.4)	10 (38.5)	10 (40.0)		

The three groups did not show significant differences in sex, age, incidence of ventilator use, length of ICU stay, Acute Physiology and Chronic Health Evaluation (APACHE II) score, total treatment time of CRRT, downtime of CRRT and underlying disease (Table 1).

The indications of intensive CRRT are shown in Table 2.

**Table 2.** Indications of continuous renal replacement therapy (CRRT).

Indications	Group 1 (*n* = 22)	Group 2 (*n* = 26)	Group 3 (*n* = 25)
	*N* (%)		
Acute kidney injury (AKI) with hyperkalemia	6 (27.3)	3 (11.5)	6 (20.0)
AKI with metabolic acidosis	11 (50.0)	14 (53.8)	13 (52.0)
AKI with hyperkalemia and metabolic acidosis	3 (13.6)	4 (15.4)	4 (16.0)
AKI with hypercatabolic state	2 (9.1)	2 (7.7)	1 (4.0)
AKI with others	0 (0.0)	3 (11.5)	1 (4.0)

The three groups also did not show a significant difference in laboratory variables prior to CRRT. Specifically, the mean serum P levels prior to CRRT were 1.64 ± 0.58, 1.56 ± 0.54, and 1.39 ± 0.75 mmol/L in groups 1, 2, and 3, with no significant difference between groups (Table 3).

**Table 3.** Biochemistry profiles prior to continuous renal replacement therapy (CRRT).

Parameter	Group 1 (*n* = 22)	Group 2 (*n* = 26)	Group 3 (*n* = 25)	*F*	*p*	
P (mmol/L)	1.64 ± 0.58	1.56 ± 0.54	1.39 ± 0.75	1.96	.16	
Ca (mmol/L)	1.99 ± 0.28	2.04 ± 0.52	1.93 ± 0.31	0.26	.76	
K (mmol/L)	4.87 ± 1.13	4.50 ± 0.92	4.58 ± 0.84	1.50	.23	
Na (mmol/L)	134.45 ± 7.23	134.11 ± 7.45	136.33 ± 5.96	1.44	.24	
Blood urea nitrogen (mg/dL)	64.52 ± 32.62	63.94 ± 27.22	52.56 ± 24.53	1.46	.24	
Cr (mg/dL)	4.20 ± 2.12	3.70 ± 1.91	3.91 ± 2.14	0.59	.62	
Hb (g/dL)	10.57 ± 1.90	10.15 ± 1.72	9.94 ± 2.09	0.91	.43	
Hct (%)	31.10 ± 5.40	29.83 ± 5.04	29.11 ± 5.54	0.70	.40	
PLT (×10^3^/µL)	111.70 ± 71.76	90.25 ± 71.33	120.11 ± 63.84	1.21	.29	

In all groups, serum phosphorus levels decreased after initiation of CRRT. Thirteen (59.0%) of 22 patients in group 1 had hypophosphatemia episodes during CRRT therapy. The incidence of mild and moderate hypophosphatemia was 54.5% and 45.5%, respectively. The onset of hypophosphatemia from the initiation of CRRT was, on average, 2.63 ± 2.44 days in group 1, 2.04 ± 2.16 days in group 2, and 2.24 ± 2.45 days in group 3; no significant difference was observed among the groups. At the onset of hypophosphatemia, the mean serum P level was 0.72 ± 0.11, 0.69 ± 0.12, and 0.72 ± 0.12 mmol/L in groups 1, 2, and 3. The serum P levels at the point of first occurrence of hypophosphatemia were approximately 0.82 ± 0.66 mmol/L lower than the baseline levels prior to CRRT in all groups.

The serum P level after correction of hypophosphatemia was 1.24 ± 0.37 mmol/L and 1.44 ± 0.31 mmol/L in group 2 and group 3, respectively, and was significantly lower in group 2 (*p* = .02). The time required for correcting hypophosphatemia with phosphate supplementation was 1.65 ± 0.80 days for group 2 and 1.39 ± 1.43 days for group 3 and was significantly longer in group 2 than group 3 (*p* = .02) (Table 4).

**Table 4.** Patterns of hypophosphatemia during continuous renal replacement therapy (CRRT).

	Group 1 (*n* = 22)	Group 2 (*n* = 26)	Group 3 (*n* = 25)	*X*^2^*/F*	*p*
	*N* (%)/mean ± standard deviation				
Serum P levels before CRRT (mmol/L)	1.64 ± 0.58	1.56 ± 0.54	1.39 ± 0.75	1.96	.16
Serum P levels at onset of hypophosphatemia (mmol/L)	0.72 ± 0.11	0.69 ± 0.12	0.72 ± 0.12	0.39	.66
ΔP*	0.92 ± 0.47	0.87 ± 0.42	0.67 ± 0.63	0.48	.78
Onset time of hypophosphatemia during CRRT (days)	2.63 ± 2.44	2.04 ± 2.16	2.24 ± 2.45	0.62	.54
Serum P levels after correction of hypophosphatemia (mmol/L)	N/A	1.24 ± 0.37	1.44 ± 0.31	4.86	.02
The time required for correcting hypophosphatemia (days)	N/A	1.65 ± 0.80	1.39 ± 1.43	6.22	.02

* ΔP: Serum P levels before CRRT – Serum P levels at onset of hypophosphatemia.

**Figure 1.**
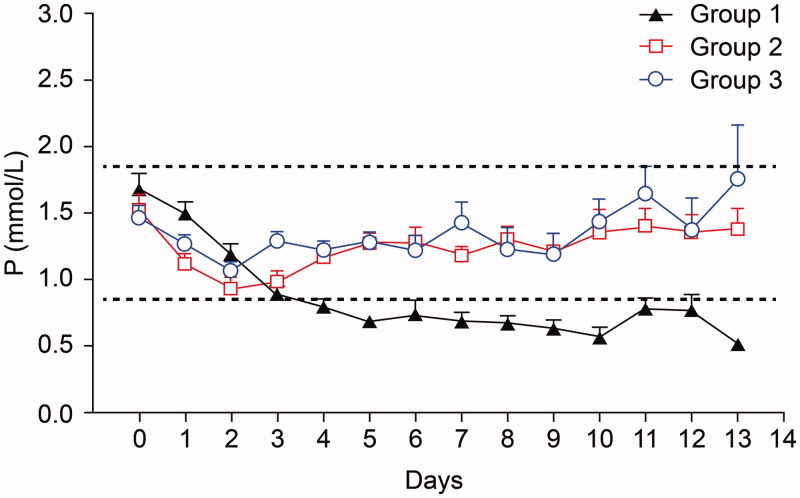
Changes in serum P level during CRRT.

After phosphate supplementation, hypophosphatemia developed in 2/26 (7%) and hyperphosphatemia occurred in 2/26 (7%) in group 2. No hypophosphatemia was seen in group 3, but hyperphosphatemia developed in 5/25 (20%). When hyperphosphatemia occurred following phosphate supplementation, the serum P level was not statistically different between groups 2 and 3 (2.00 ± 0.08 vs. 2.06 ± 0.06 mmol/L, *p* = .54). When phosphate supplementation was stopped due to hyperphosphatemia, the level returned to normal within 2.0 days in group 2 and 1.0 day in group 3 (*p* = .17), and there was no statistically significant difference in serum P levels between the groups (group 2 *vs.* 3; 1.53 ± 0.28 *vs.* 1.20 ± 0.14 mmol/L, *p* = .23). The overall mean serum P level during the total duration of CRRT was significantly different across the three groups at 0.88 ± 0.34, 1.22 ± 0.16, and 1.36 ± 0.17 mmol/L in groups 1, 2, and 3, (*p* < .0001) (Figure 1).

**Figure 2.**
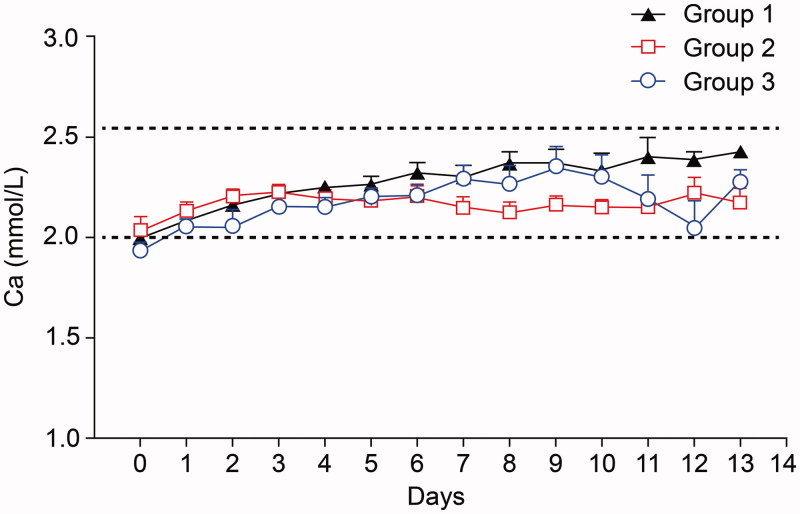
Changes in serum Ca level during CRRT.

The mean serum Ca level before initiation of CRRT was 1.99 ± 0.29, 2.04 ± 0.29, and 1.92 ± 0.29 mmol/L in groups 1, 2, and 3, and these were not statistically different (*p* = .77). However, the overall mean serum Ca level during CRRT was statistically different among the three groups (2.78 ± 0.13, 2.17 ± 0.05, and 2.18 ± 0.12 mmol/L in groups 1, 2, and 3, *p* = .01) (Figure 2).

**Figure 3.**
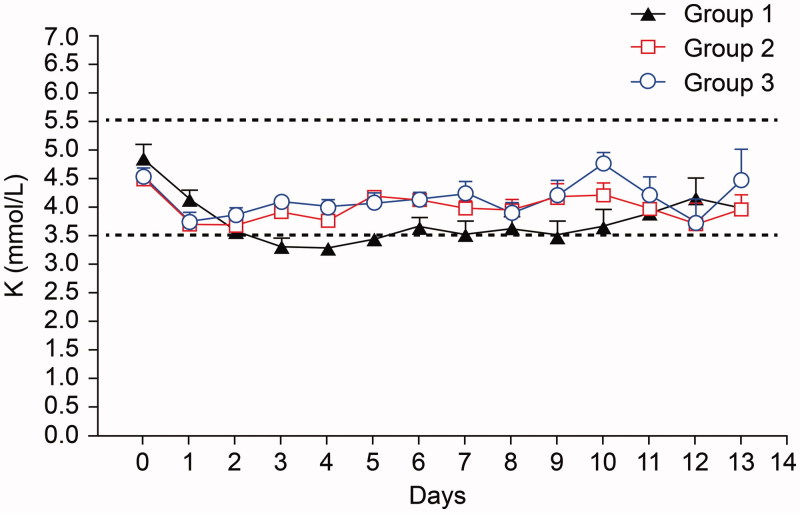
Changes in serum K level during CRRT.

Serum K level was not statistically different among the groups either before initiation of CRRT (*p* = .40) or during CRRT (*p* = .07). The mean serum K level prior to CRRT was 4.88 ± 1.17, 4.49 ± 0.61, and 4.52 ± 0.75 mmol/L in groups 1, 2, and 3, and the overall mean serum K level during CRRT was 3.86 ± 0.43, 3.99 ± 0.23, and 4.14 ± 0.30 mmol/L in groups 1, 2, and 3 (Figure 3).

## Discussion

The results of this study demonstrated that hypophosphatemia frequently occurred during intensive CRRT and could be effectively corrected by the addition of phosphate supplementation to the dialysate and replacement solutions at a concentration of 2.0 mmol/L or 3.0 mmol/L. CRRT is highly effective in urea clearance but commonly causes hypophosphatemia because it clears phosphate simultaneously. In particular, a higher effluent flow rate and a prolonged period of CRRT increase the incidence of CRRT-induced hypophosphatemia [14–16]. In the present study, hypophosphatemia occurred approximately 2.30 days after CRRT was initiated. The mean serum phosphorus level at the time when hypophosphatemia first occurred was approximately 0.71 mmol/L, which was an average decrease of 0.82 mmol/L from the pre-CRRT level. The incidence of hypophosphatemia was approximately 59.0%, showing a discrepancy from existing studies. A multi-center RCT conducted with 1508 patients (the RENAL Replacement Therapy Study) reported an incidence of hypophosphatemia of 65% in the higher-intensity CRRT group (an effluent flow of 40 mL/kg/hr) [17], an incidence higher than the current study finding. However, in another large-scale multi-center study (the ATN study) the incidence of hypophosphatemia was 17.6% in the intensive therapy group, which is lower than the result of the present study [18]. It is speculated that the incidence of hypophosphatemia in the present study was different from the findings of the large-scale RCTs due to variations in the patient populations and prescribed doses of CRRT. The RENAL study, in contrast to the present study, included patients with post-dilution continuous venovenous hemodiafiltration (CVVHDF) only. In addition, the dose prescribed for the high-intensity group in the RENAL study was 40 mL/kg/hr, higher than in the present study. In the ATN study, the prescribed dose for the high-intensity group was 35 mL/kg/hr, the same as in the present study, however, the group included not only patients with pre-dilution CVVHDF but also those undergoing intermittent hemodialysis or sustained low-efficiency dialysis. These differences are believed to have caused the incidence of hypophosphatemia in the present study to be lower, compared to the RENAL study, and higher compared to the ATN study.

Despite the difference in incidence across the studies, a common conclusion is that hypophosphatemia is more likely to occur at a high CRRT dose. In the RENAL study, the incidence of hypophosphatemia was significantly higher in the higher-intensity CRRT group (with an effluent flow of 40 mL/kg/hr) than in the lower-intensity CRRT group (25 mL/kg/hr) (65% *vs.* 54%, *p* < .001). In the ATN study too, the incidence was significantly higher in the intensive therapy group compared to the less-intensive therapy group (17.6% *vs.* 10.9%, *p* = .001). Accordingly, the occurrence of hypophosphatemia during intensive CRRT should receive more attention.

On the other hand, it is common for critically ill patients to receive intensive CRRT. Earlier studies reported that 35 mL/kg/hr of ultrafiltration (in post-dilution) had a positive impact on survival compared with 20 mL/kg/hr of ultrafiltration [22]. However, several RCTs that compared intensive and less intensive CRRT failed to observe a clear beneficial effect of a higher dose on mortality or kidney function recovery [17,18]. The ATN study compared standard-intensity predilution CRRT (a prescribed effluent flow of 20 mL/kg/hr) and a high-intensity CRRT (35 mL/kg/hr) and did not find a between-group difference in the outcome. The RENAL Study compared a conventional dose post-dilution CRRT (25 mL/kg/hr) and a high-intensity CRRT (40 mL/kg/hr), and there was no difference between the two groups. These findings are the evidence suggesting that increasing CRRT dose to effluent flow higher than 25 mL/kg/hr does not provide additional benefits like reduced mortality. Hence, The Kidney Disease: Improving Global Outcome (KDIGO) Clinical Practice Guideline for AKI has recommended a delivered effluent flow of 20–25 mL/kg/hr [19] . A treatment downtime, however, is inevitable in clinical practice whether it is due to a scheduled or unscheduled interruption. Thus, the delivered dose is always lower than the prescribed dose. Indeed, in the RENAL study, the delivered dose was decreased by 12–16% compared to the prescribed dose [17]. Therefore, to ensure a delivered dose of 20–25 mL/kg/hr, it may be necessary to prescribe approximately 25–30 mL/kg/hr, and it is also necessary to minimize CRRT downtime to 4 h per day or less.

In the above-mentioned several clinical trials, it has been demonstrated that increasing CRRT dose does not improve the survival rate of a critically ill patient with AKI. However, this finding does not necessarily imply that CRRT dose is not important. Some patients may require a higher CRRT dose than generally recommended. They include critically ill hypercatabolic patients (e.g., burn injury or tumor lysis syndrome), patients who need an urgent correction of severe hyperkalemia or metabolic acidemia, and rhabdomyolysis patients in whom myoglobin should be rapidly removed [21]. Particularly in postsurgery AKI patients, high-intensity CRRT reduces the risk of death [20]. In other words, CRRT should not be administered uniformly at a fixed dose of 20–25 mL/kg/hr in all patients. Rather, the dose should be determined dynamically according to patient status. In 2016, the Acute Disease Quality Initiative (ADQI) Consensus Group proposed that CRRT doses should be dynamic, in recognition of between- and within-patient variation in targeted solute control or unintended solute clearance [21]. Regardless of the reason for administering high-intensity CRRT, hypophosphatemia is more likely to occur in comparison to less intensive CRRT, and it is associated with poor outcome, including prolonged respiratory failure. Therefore, appropriate approaches to prevent and treat CRRT-induced hypophosphatemia are necessary.

In general, hypophosphatemia is corrected by phosphate supplementation in patients who complain of symptoms or show severe hypophosphatemia [23]. Recently, however, it has been reported that even mild hypophosphatemia is associated with poor outcome. Suzuki et al. reported that hypophosphatemia <0.6 mmol/L increased the incidence and duration of mechanical ventilation [24]. Demirjian et al. found that serum phosphorus <2 mg/dL (0.67 mmol/L) during CRRT was associated with an increased need for tracheostomy [3]. Lim et al. reported that the risk of prolonged mechanical ventilation (≥7 days) was increased even in patients whose serum phosphorus was <2.9 mg/dL (0.94 mmol/L) during CRRT [adjusted OR 14.0, 95% CI (1.37, 143.90), *p* = .03] [7]. Therefore, aggressive prevention and treatment is required even in mild hypophosphatemia during CRRT. The present study examined patients undergoing high-intensity CRRT with the prescribed dose of 35 mL/kg/hr for various reasons. Accordingly, the study is of significance in that it proposed an approach of CRRT-induced hypophosphatemia treatment in patients undergoing intensive CRRT for reasons including postsurgery AKI.

There are several approaches for phosphate repletion to prevent hypophosphatemia in CRRT patients. Phosphate may be administered via the oral, enteric, or intravenous route. During CRRT, through intravenous supplementation, it is not easy to maintain serum phosphorus at a stable level or to predict the change in serum phosphorus level. Recently, phosphate is mainly added to dialysate and replacement fluids [9–11]. Santiago et al. found that the addition of 0.8 mmol/L phosphate to replacement and dialysate solutions reduces the incidence of hypophosphatemia and the need for intravenous phosphate treatment in pediatric patients [10]. Troyanov et al. reported that the addition of 1.2 mmol/L of phosphate to the dialysis fluid and the replacement fluid could eliminate the episodes of hypophosphatemia in adult CRRT patients [11]. Recently, it was reported that commercially prepared phosphate-containing solutions such as Phoxilium^®^ are effective in the prevention of hypophosphatemia in CRRT patients [12,25,26].

Even with such phosphate repletion regimens, hypophosphatemia during CRRT cannot be completely prevented. Especially in the case of intensive CRRT with a high effluent flow rate, it is difficult to avoid CRRT-induced hypophosphatemia. Broman et al. reported that a hypophosphatemic episode did not occur when CRRT was administered with Phoxilium^®^ (phosphate 1.2 mmol/L) as dialysate and replacement fluid. However, the effluent flow rate was only 18 mL/kg/hr in their study [4]. Pistolesi et al. observed that when CRRT was administered at a prescribed dialysis dose of 27.0 mL/kg/hr, prolonged use of Phoxilium^®^ was able to induce and/or maintain normophosphatemia in 88% of the patients after 72 h of CRRT treatment, and in the whole treatment period, only 4.6% of serum phosphorus determinations met the criteria for mild or moderate hypophosphatemia [27]. In contrast, Lim et al. found that hypophosphatemia occurred in 29.5% of patients undergoing CRRT with an effluent fluid rate of 33 mL/kg/hr, even if 1.0 mmol/L phosphate was added to dialysate and replacement fluids [7]. Accordingly, during intensive CRRT, phosphate should be supplemented at a level much higher than commercialized phosphate-containing solutions to prevent and treat hypophosphatemia. In the present study, we added 2 mmol/L (group 2) or 3 mmol/L (group 3) of phosphate instead of 1.2 mmol/L either to the dialysate or replacement solutions in critically ill patients receiving CRRT with 35 mL/kg/hr of effluent flow rate. Following the supplementation of 2 mmol/L or 3 mmol/L of phosphate, hypophosphatemia was effectively corrected in both the groups, with low rates of subsequent hyperphosphatemia and hypophosphatemia in either group. In the present study, hyperphosphatemia was neither severe nor associated with poor outcome. However, like hypophosphatemia, hyperphosphatemia can negatively affect the prognosis of CRRT patients [28], and hence, the level of phosphate added during CRRT should differ depending on the serum phosphorus level.

The present study demonstrates the safety and efficacy of phosphate supplementation added to dialysate and replacement fluids to treat hypophosphatemia in patients requiring intensive CRRT. However, this was a relatively small, retrospective study. As such, the conclusions of statistical significance testing are limited. In addition, it was not possible to obtain information on the delivered dose from the EMR, and this would have been useful in comparison to the prescribed dose. Although the study demonstrated that phosphate supplementation may effectively treat hypophosphatemia once it occurs, phosphate containing solution was not administered from the initiation of CRRT, and prevention of hypophosphatemia was not demonstrated. Further, nutritional supplementation which could impact the occurrences of hypophosphatemia and hyperphosphatemia were not analyzed. To overcome these limitations, a large randomized prospective study should be conducted. In addition, this treatment approach should be applied with caution in practice.

## Conclusion

The addition of 2 mmol/L phosphate to dialysate and replacement solution effectively corrected hypophosphatemia during intensive CRRT administered with an effluent flow rate of 35 mL/kg/h and stabilized serum phosphorus levels. The present study suggests considerations for determining the optimized level of phosphate supplementation in the dialysate and replacement fluids. The use of 2 mmol/L phosphate is a valid method in hypophosphatemic patients, but a different concentration of phosphate could be more appropriate if the aim is to prevent hypophosphatemia at the start of CRRT.

## Supplementary Material

Supplementary Table

## Data Availability

The authors confirm that the data supporting the findings of this study are available within the article.

## Geolocation information

Seoul, Korea.
