# Supplementary material for: Phosphate supplementation for hypophosphatemia during continuous renal replacement therapy in adults
Source: Ren Fail. 2019 Mar 26;41(1):72–9. doi: 10.1080/0886022X.2018.1561374 (PMC6442196; doi:10.1080/0886022X.2018.1561374)
Supplement: Supplementary Table [file IRNF_A_1561374_SM2593.pdf]

Supplementary Table 1. Compositions of Hemosol B0 used for dialysate and replacement solutions during continuous renal replacement therapy

Composition	mmol/L
Sodium, Na^{++}	140
Calcium, Ca^{++}	1.75
Magnesium, Mg^{++}	0.50
Chloride, Cl^{++}	109.5
Lactate, $C_3H_5O_3^-$	3
Bicarbonate, HCO_3^-	32
Potassium, K^+	0
